# Selective Laser Sintering 3D Printing of Orally Disintegrating Printlets Containing Ondansetron

**DOI:** 10.3390/pharmaceutics12020110

**Published:** 2020-01-30

**Authors:** Nour Allahham, Fabrizio Fina, Carmen Marcuta, Lilia Kraschew, Wolfgang Mohr, Simon Gaisford, Abdul W. Basit, Alvaro Goyanes

**Affiliations:** 1 FabRx Ltd., 3 Romney Road, Ashford, Kent TN24 0RW, UK; nour.ahham.17@ucl.ac.uk (N.A.); s.gaisford@ucl.ac.uk (S.G.); 2 Department of Pharmaceutics, UCL School of Pharmacy, University College London, 29–39 Brunswick Square, London WC1N 1AX, UK; fabrizio.fina.14@ucl.ac.uk; 3 Losan Pharma GmbH, Otto-Hahn-Strasse 13, 79395 Neuenburg, Germany; carmen.marcuta@losan.de (C.M.); lilia.kraschew@losan.de (L.K.); wolfgang.mohr@losan.de (W.M.); 4 Departamento de Farmacología, Farmacia y Tecnología Farmacéutica, I+D Farma Group (GI-1645), Universidade de Santiago de Compostela, 15782 Santiago de Compostela, Spain

**Keywords:** three-dimensional printing, 3D printed drug products, printing pharmaceuticals, additive manufacturing, rapid prototyping, orally disintegrating tablets (ODTs), orally disintegrating printlets (ODPs), taste masking, personalized medicines

## Abstract

The aim of this work was to explore the feasibility of using selective laser sintering (SLS) 3D printing (3DP) to fabricate orodispersable printlets (ODPs) containing ondansetron. Ondansetron was first incorporated into drug-cyclodextrin complexes and then combined with the filler mannitol. Two 3D printed formulations with different levels of mannitol were prepared and tested, and a commercial ondansetron orally disintegrating tablet (ODT) product (Vonau^®^ Flash) was also investigated for comparison. Both 3D printed formulations disintegrated at ~15 s and released more than 90% of the drug within 5 min independent of the mannitol content; these results were comparable to those obtained with the commercial product. This work demonstrates the potential of SLS 3DP to fabricate orodispersible printlets with characteristics similar to a commercial ODT, but with the added benefit of using a manufacturing technology able to prepare medicines individualized to the patient.

## 1. Introduction

Ondansetron is an anti-emetic drug, listed on the World Health Organisation (WHO) List of Essential Medicines, which is used as the first-line therapy for chemotherapy- and radiation-induced nausea and vomiting with a dose of 16 mg daily [1]. Ondansetron is commercially available as soluble films or orally disintegrating tablets (ODT) due to its low solubility in water and to minimise water intake, which can induce vomiting [2]. However, one of the challenges for delivering ondansetron in the mouth is its bitter taste. Different taste-masking and formulation strategies have been reported, including the use of sweeteners, ion-exchange resins [3], superdisintegrants such as crospovidone [2], glycine-chitosan mixtures [4] or enteric polymers such as Eudragit [5].

A family of taste masking excipients often used are cyclodextrins. They are cyclic oligosaccharides that can encapsulate hydrophobic drugs into their cavity while having a hydrophilic outer surface [6,7]. The formation of these inclusion complexes helps to improve the physiochemical properties of hydrophobic drugs, increasing their water solubility, bioavailability and stability [6]. The ability of cyclodextrins to form such complexes is also exploited for taste-masking purposes of oral medicines, which could tackle the bitter-taste of ondansetron. Cyclodextrin-drug inclusion complexes can be easily prepared in different ways, with co-precipitation being the most common [8]. In this method, the cyclodextrin is dissolved in an appropriate solvent such as ethanol, and the drug is then added gradually under continuous stirring until evaporation of the solvent. The drug-cyclodextrin complexes may be also formed “in situ” in the mouth facilitated by the saliva as a solvent [6].

Despite the high safety profile of ondansetron, one of its adverse effects is arrhythmia and dose-dependent QT-interval elongation when given with other medications, which requires monitoring and control of dose [9]. QT-interval elongation is a life-threatening arrhythmia, which can be induced by many drugs and lead to sudden cardiac death. For vulnerable populations such as cancer patients or the elderly taking different medicines, the use of personalised and dose-specific dosage forms is desirable.

Three-dimensional printing (3DP) is an innovative additive manufacturing technology that has come to the fore in the preparation of personalised dose printlets (3D printed tablets) [10,11,12,13,14]. 3D printing is an umbrella term that encompasses various technologies, many of which have already been evaluated in the pharmaceutical field [15,16,17,18], including powder bed inkjet printing, fused deposition modelling (FDM) [19,20,21,22,23,24,25,26,27], semi-solid extrusion (SSE) [28,29,30,31,32,33], selective laser sintering (SLS), direct powder extrusion (DPE) [34] and stereolithography (SLA) [15,35,36,37,38].

Selective laser sintering (SLS) is one of the latest and most advanced technologies proposed for the preparation of solid dosage forms [39]. SLS is a one-step fabrication process involving a laser to selectively sinter powder particles in a layered manner to form 3D structures. The SLS printer consists of a powder bed, a powder reservoir, a roller and a laser source. The powder for printing is homogenously spread on the powder bed by the roller. Depending on the 3D design of the object, the laser is focused to draw specific patterns on the powder surface sintering and agglomerating the powder particles. Once the first layer is sintered, the powder bed moves down while the reservoir bed moves up to allow for the delivery of a new layer of powder on top of the previous one. The technology was originally designed to print objects at high temperatures using metallic, ceramic or thermoplastic materials like PA12 (Nylon) or PEEK (Polyether ether ketone) [40]. For a while, SLS printing technology was not considered suitable for the preparation of medicines due to the potential degradation of the drugs caused by the high energy of the CO_2_ lasers that work in the IR region of the spectra [15]. Nowadays, however, the use of SLS printers that use lower intensity diode lasers has made it possible to fabricate novel drug products with no drug degradation [41]. In the pharmaceutical field, SLS 3D printing has been recently used to prepare different types of printlets [42], miniprintlets [43], drug delivery lattice structures [44], drug delivery devices [45] and formulations in the form of films and printlets for quick dose verification using a rapid point-and-shoot approach [46].

One of the potential applications of SLS technology is its ability to fabricate orally disintegrating tablets (ODTs) [47]. The definition of ODTs in the European Pharmacopoeia defines these systems as oral dosage forms that disintegrate in less than 3 min [48], while according to the Food and Drug Administration (FDA), they are oral dosage forms that dissolve within 30 s when in contact with saliva [49]. The accelerated disintegration of ODTs enhances the bioavailability and absorption of drugs [50,51], and ODTs are more appropriate for patients with dysphagia or those who have difficulties in swallowing e.g., children and elderly. A variety of methods are available to produce ODTs such as freeze-drying, spray drying and direct compression. These methods, however, are limited by high manufacturing costs and complexity, therefore, more cost-effective methods are required [52,53].

The aim of this study was to develop new orally disintegrating printlets (ODPs) incorporating ondansetron-cyclodextrin complexes using SLS 3D printing. Mannitol was included as a filler due to its taste masking properties. The performance of the 3D printed formulations were evaluated and compared to a commercial ODT formulation of ondansetron (Vonau^®^ Flash 8 mg).

## 2. Materials and Methods

### 2.1. Materials

Ondansetron Hydrochloride USP grade was obtained from Sun Pharma, Mumbai, India (MW 293 g/mol), β-Cyclodextrin Cavamax^®^ W7 (MW 1135 g/mol) was obtained from Ashland, Ashland, Düsseldorf, Germany. Kollidon^®^ VA-64 (vinylpyrrolidone-vinyl acetate copolymers) was obtained from BASF, London, UK. Candurin^®^ Gold Sheen was purchased form Azelis, Hertford, UK. Mannitol Parteck^®^ Delta M was obtained from Merck, Darmstadt, Germany.

The commercial medicine tested in this study is Vonau^®^ Flash (Biolab, Brasil), an ODT ondansetron hydrochloride formulation. Its composition includes mannitol, microcrystalline cellulose, crospovidone, magnesium stearate, silicon dioxide, strawberry flavour and aspartame. The product is available in two strengths, 4 mg and 8 mg ondansetron.

### 2.2. Preparation of 1:5 Ondansetron: Cyclodextrin Complex

20 g of cyclodextrin powder was dissolved in 40 mL of ethanol, then 4 g of ondansetron was added gradually under continuous stirring. The wet mixture was then placed in an oven at 40 °C until the evaporation of the ethanol.

### 2.3. 3D Printing Process

A mortar and pestle was used to blend 100 g of a combination of the drug-cyclodextrin complex and excipients (Table 1). In order to enhance energy absorption from the laser and facilitate printability 3% of Candurin^®^ Gold Sheen (colorant) was incorporated into the formulations. The final mixture of materials were then placed into a Desktop SLS printer (Sintratec Kit, AG, Brugg, Switzerland) to prepare the oral dosage forms. Cylindrical printlet templates were designed with AutoCAD 2014 (Autodesk Inc., San Rafael, Ca., USA) to obtain an ondansetron dose of 8 mg in the 3D printed formulations (12.4 mm diameter × 3.6 mm height). 3D templates were transferred as a STL format files into the 3D printer Sintratec central software Version 1.1.13 (Sintratec Kit, AG, Brugg, Switzerland).

The powder mixture of excipients and drug was transferred by a sled from the platform that contains the powder reservoir to the building platform of the printer creating a flat layer of material [54]. The parameters surface temperature (100 °C) and chamber printing temperatures (80 °C) were kept constant. The diode laser (445 nm, blue laser, 2.3 W) sintered the powder on to the building platform following a particular arrangement based on the 3D model design (laser scanning speed 200 mm/s). Then, the reservoir platform moved up, the building platform moved down, and the sled delivered a thin layer (100 µm) of material on top of the previous layer. This procedure was reiterated layer-by-layer until the object was finished. At the end, the printlets were separated from the powder and the excess powder was removed. Printlets of each formulation were printed in batches of 6.

### 2.4. Thermal Analysis

Differential scanning calorimetry (DSC) was used to characterise the powders and drug-loaded 3D printed formulations (Q2000 DSC, TA instruments, Waters, LLC, New Castle, DE, USA) (heating rate 10 °C/min, purge gas (Nitrogen) flow 50 mL/min). The calibration for cell constant and enthalpy was done with indium (Tm = 156.6 °C, ∆Hf = 28.71 J/g) according to the manufacturer’s instructions. Aluminium (TA) pans and lids (Tzero) were used with an average sample mass of 7–9 mg. TA Advantage software (version 2.8.394) and TA Instruments Universal Analysis 2000 were used to collect and analyse the data, respectively.

### 2.5. X-ray Powder Diffraction (XRPD)

Circular 3D printed films (23 mm diameter × 1 mm height) obtained from the mixtures of drug and excipients were prepared and analysed. Raw ondansetron powder and the powder mixtures were also tested. The X-ray powder diffraction data was obtained using a Rigaku MiniFlex 600 (Rigaku Europe, UK) with a Cu Kα X-ray source (λ = 1.5418Å). Intensity 15 mA, voltage applied 40 kV, angular range of data acquisition 3–60° 2θ, stepwise size of 0.02°, speed of 5°/min.

### 2.6. Characterisation of the Printlets

#### 2.6.1. Determination of Printlet Morphology

The diameter and thickness of the printlets were measured using a digital calliper. Pictures of the printlets were taken with a camera Nikon Coolpix S6150 (Nikon, Tokyo, Japan) with the macro option of the menu.

#### 2.6.2. Determination of the Mechanical Properties of the Printlets

The breaking force of each printlet type (*n* = 6) was determined using a tablet hardness tester TBH 200 (Erweka GmbH, Heusenstamm, Germany). An increasing force was applied perpendicular to the formulation axis from opposite sides of a printlet until it breaks.

#### 2.6.3. Scanning Electron Microscopy (SEM)

A scanning electron microscope (SEM, JSM-840A Scanning Microscope, JEOL GmbH, Tokyo, Japan,) was used to take images of the surface and cross-section of the printlets. A thin layer of carbon (~30–40 nm) was used to coat all the samples.

#### 2.6.4. X-ray Micro Computed Tomography (Micro-CT)

A X-ray microcomputed tomography scanner (SkyScan1172, Bruker-microCT, Billerica, MA., USA) examined the internal structure, density and porosity of the 3D printed formulations (scanner resolution: 2000 × 1048 pixels). 3D imaging was completed by rotating the object through 180° with steps of 0.4° and 4 images were recorded at each step. NRecon software (version 1.7.0.4, Bruker-microCT, Bruker-microCT, Billerica, MA, USA) was used for image reconstruction. 3D model rendering and viewing were completed using the software CT-Volume (CTVol version 2.3.2.0, Bruker-microCT, Billerica, MA, USA). The data was analysed with the software CT Analyzer (CTan version 1.16.4.1, Bruker-microCT, Billerica, MA, USA). The density of the printlets was indicated with different colours. Porosity values were calculated using the 3D analysis in the morphometry preview (100 layers were chosen and evaluated at the top, central and bottom part of the printlets).

#### 2.6.5. Determination of Drug Content by High-performance Liquid Chromatography (HPLC)

Printlets of each formulation (n=2) were dissolved in volumetric flasks containing HPLC water (100 mL). Samples of the solution were filtered through a 0.4 µm filter (Millipore Ltd., Cork, Ireland) and the drug concentration quantified by HPLC (Hewlett Packard 1050 Series HPLC system, Agilent Technologies, London, UK). Injecting volume: 20 µL, mobile phase A: NaH_2_PO_4_ buffer (30%) and mobile phase B: Acetonitrile (70%), column: Eclipse Plus C18 5 µm, size: 250 × 4.6 mm (Restek, State College, PA, USA), temperature: 30 °C, flow rate: 1 mL/min, wavelength: 216 nm.

For determination of impurities, HPLC analysis was performed according to USP monograph for ondansetron [55].

A buffer solution was prepared by dissolving 2.8 g NaH_2_PO_4_·H2O in 1.00 L of HPLC water. The pH was adjusted to 5.40 (±0.05) with a sodium hydroxide solution. Mobile phase A was prepared by mixing 0.85 L buffer solution with 0.15 L acetonitrile. Mobile phase B was prepared by mixing 0.60 L buffer solution with 0.40 L acetonitrile. Formulations were dissolved into a 20 mL volumetric flask and 2 mL hydrochloric acid (0.1 M) was added. Then the volume was adjusted with mobile phase A to 20 mL. Samples of solution were then filtered through 0.45 µm filters (Millipore Ltd., Ireland) and degradation products assessed with high-performance liquid chromatography (HPLC) (Agilent 1100 Series HPLC system, Agilent Technologies, Germany) by an external standard method. The validated HPLC method entailed injecting 20 µL samples for analysis using a gradient of mobile phase A and mobile phase B (0 min: 100% mobile phase A; 20 min: 100% mobile phase B; 22 min: 100% mobile phase B; 23 min: 100% mobile phase A until 30 min) through a spherical nitrile silica gel 5 µm column, 250 × 4.6 mm (Waters, Germany) maintained at 20 °C. The mobile phase was pumped at a flow rate of 1.5 mL/min and the eluent was screened at a wavelength of 216 nm. The limit of quantification was 0.05%. Analysis was performed in triplicate. The mean values are reported.

#### 2.6.6. Disintegration Testing Conditions

Disintegration tests of the commercial formulation and the printlets were conducted using a USP disintegration apparatus. The basket was filled with 650 mL of water at 37 ± 0.5 °C. One printlet was gently placed in each tube and disks were placed. The time for the printlet to completely disintegrate was then observed. Six printlets of each formulation were evaluated.

#### 2.6.7. Dissolution Testing Conditions

Drug dissolution profiles for the commercial and the 3D printed formulations were obtained with a USP-II apparatus (Model PTWS, Pharmatest, Germany). The formulations were placed in 500 mL of 0.1 M HCl, as indicated in the USP monograph for ondansetron ODT. USP-II was fixed at a paddle speed of 50 rpm and at a temperature of 37 ± 0.5 °C (*n* = 3). An in-line UV spectrophotometer was used to determine the percentage of drug released from the printlets (Cecil 2020, Cecil Instruments Ltd., Cambridge, UK) at 310 nm using Icalis software (Icalis Data Systems Ltd., Berkshire, UK).

## 3. Results and Discussion

Two different formulations incorporating different percentages of mannitol and the polymer Kollidon VA-64 were initially tested to assess their printability (Table 1). The excipients were selected with the aim of producing accelerated drug release formulations, with ultimately, the objective of fabricating printlets with orally disintegrating characteristics. The formulations incorporated the ondansetron as drug-cyclodextrin complexes to facilitate drug dissolution and to provide potential taste masking properties. The presence of cyclodextrin did not affect the sintering process even though the particle size of the complexes was not controlled.

The fabrication of the ODPs was successfully achieved at the laser scanning speed of 200 mm/s to obtain two different types of formulations. Different laser scanning speeds were evaluated in preliminary tests based on results from previous studies [47]. The selected scanning speed was 200 mm/s based on the mechanical properties and the dissolution characteristics of the printlets. All formulations contained 22% *w/w* of ondansetron-cyclodextrin mixture (ratio 1:5) and 3% *w/w* colorant Candurin^®^ gold sheen. Candurin^®^ gold sheen is a pharmaceutical excipient added to the formulations to facilitate the printing process [41]. As the sintering process without the use of colorants is not successful as the powder did not absorb the light at the wavelength of the laser of the printer. No interactions were observed between Candurin^®^ gold sheen and the rest of the components of the formulations.

**Figure 1:**
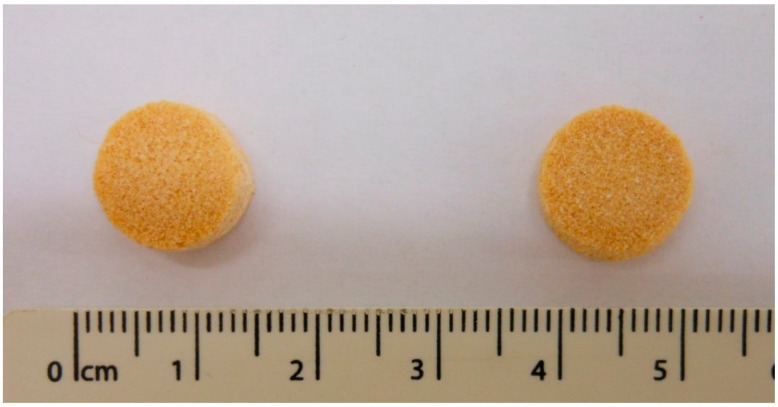
Images of the Formulation I (**left**) and Formulation II (**right**) (units are in cm).

The printlets were cylindrical in shape, and yellow in colour due to the colorant (Figure 1).

**Figure 2:**
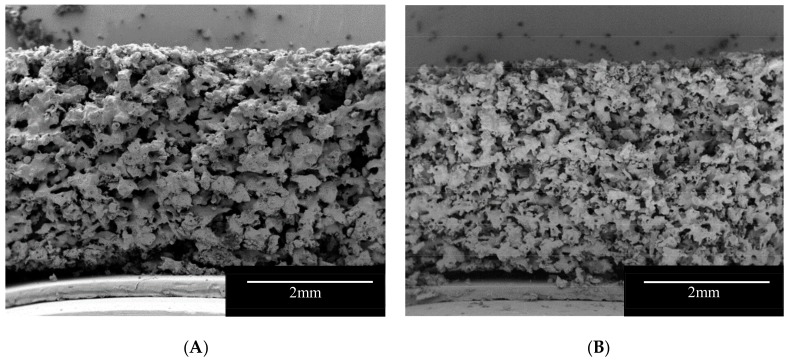
SEM Images of the vertical cross-section of the Formulation I (**A**) and II (**B**).

SEM images provided visual information on the internal structure of the printlets (Figure 2). The cross-section of the formulations showed a very porous structure that may facilitate the penetration of liquid in the formulations leading to a rapid disintegration of the printlets. At printing temperatures (100 °C) Kollidon VA-64 (Tg 101 °C, [56]) is on a rubbery state and following the passage of the laser, the polymer particles connect to each other forming bridges and sintered areas. On the other hand, mannitol has a much higher melting point (168 °C) and at printing temperatures, the powder particles partially dissolve in the rubbery Kollidon VA-64 and the rest is trapped unmodified within the polymer matrix. The fact that part of the mannitol remained in the powder form allowed the manufacture of highly porous matrix printlets with fast disintegrating properties. Additionally, mannitol is an osmotic agent [57], the presence of the osmotic sugar in the formulation, may allow the printlet to rapidly imbibe water into its core generating an internal pressure that can break apart the sintered bridges. Formulation I showed larger sintered areas due to the higher content of Kollidon VA-64 (Figure 2A). 

**Figure 3:**
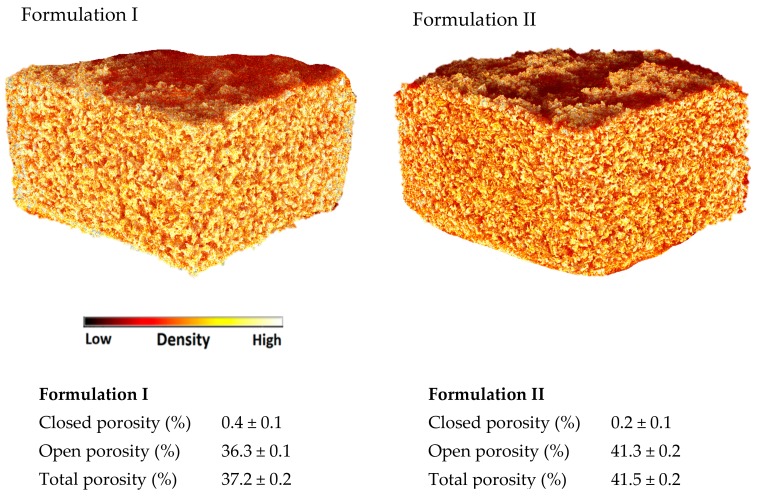
X-ray micro-CT Images of the (**Formulation I**) and (**Formulation II**).

X-ray micro-CT is a powerful tool to visualise the internal structure and density of the 3D printed formulations (Figure 3) and it can be used to calculate their porosities. Closed porosity identifies the pores of the printlets that do not have contact with the external environment. If the printlets are immersed in the dissolution medium, the medium cannot enter into the closed pores unless the medium dissolves the external walls of the pores. On the other hand, open porosity identifies the empty spaces inside the 3D printed formulations that are connected with other pores and with the external environment. In the dissolution medium a structure that contains open pores would dissolve quicker than the corresponding structure with closed pores. The sum of closed and open pores defines the total porosity. In this study, both formulations showed a negligible closed porosity but a similar high total porosity (37.2–41.5%). Formulation I showed slightly lower open porosity (36.3%) compared to Formulation II (41.3%). The difference may be due to the higher amount of Kollidon VA-64 in Formulation I that produce more sintered zones. Both formulations showed a similar density, represented as comparable in colour (Figure 3). All the small red areas in the CT image (Figure 3) represent the air (low density) indicating the presence of pores. Formulation II shows more red areas indicating a lower degree of sintering and therefore an increased porosity.

Printlets of both formulations show similar dimensions (Table 2) less than 12 mm in diameter and around 4 mm in height. Formulation II shows the closest dimension (11.97 mm diameter × 3.78 mm height) to the designed 3D model (12.4 mm diameter × 3.6 mm height). The printlets obtained from both formulations also show very similar weights around 215 mg (Table 2), which is needed for a dose of 8 mg of ondansetron.

These formulations do not break readily during manipulation and show properties appropriate for handling. Both formulations have similar breaking force values of 14.7 N for Formulation I and 18.5 N for Formulation II (Table 2). Even though the values are reduced, the fact that there is no minimum requirement for the breaking force of ODT formulations would make them suitable if they are conditioned in blister packs like most ODT formulations.

The drug loading of the printlets was quantified using HPLC, and it was very similar to the theoretical values (Table 2). Drug degradation is a problem that could have been encountered in the study, therefore, we quantified the organic degradation products of ondansetron by HPLC analysis according to the USP monograph. All detected organic degradation products of ondansetron showed impurities below 0.2% and confirmed therefore that no degradation of ondansetron occurs during SLS printing. The small difference in drug loading from the theoretical content may be explained by small variations in the distribution of the drug in the excipients and due to experimental variations. Conventional SLS printers that use CO_2_ lasers working in the IR region of the spectra may burn and modify the properties of the polymer and degrade the drugs, however, the desktop printer used in this study has a blue diode laser with lower intensity working in the blue region of the spectra. Since no degradation took place, this lower intensity together with the different wavelength has proved safe for printing the drug ondansetron.

**Figure 4:**
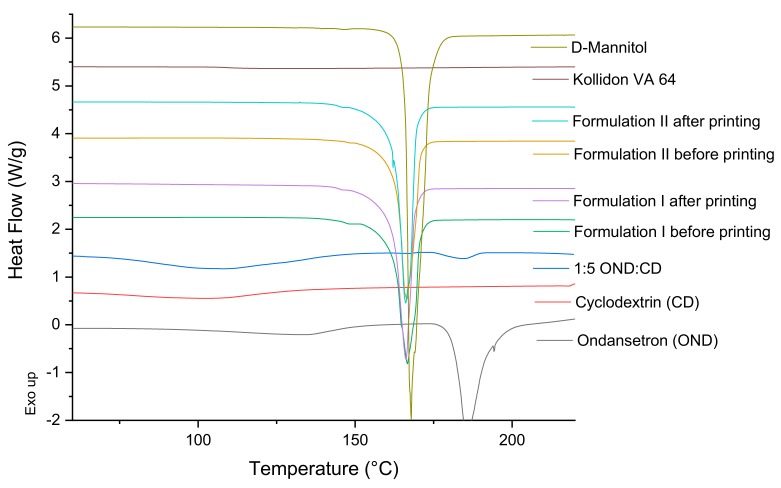
DSC thermograms of pure drug, individual polymers, powder mixtures before printing and the printlets.

**Figure 5:**
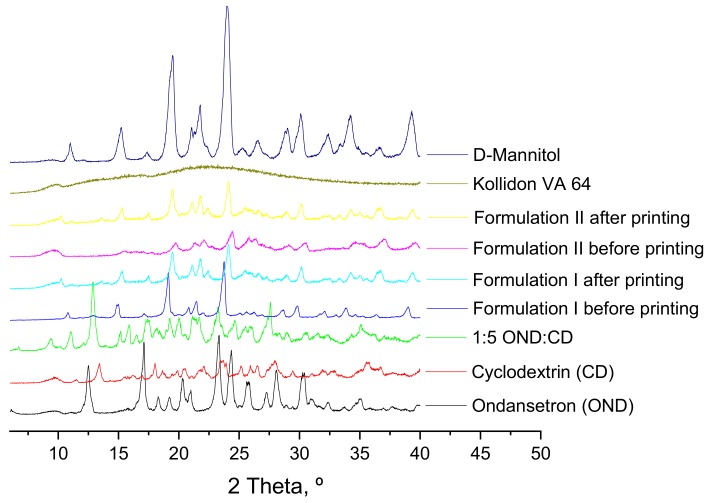
X-ray powder diffractograms of pure drug, individual polymers, powder mixtures before printing and 3DP discs.

DSC and X-ray studies of the individual components of the formulations, of 1:5 ondansetron-cyclodextrin complexes, of the mixture of the components before the printing process and of the 3D printed formulations were performed to characterize the state of the drug and how it is incorporated into the 3D printed formulations (Figure 4 and Figure 5).

DSC data of the ondansetron pure powder indicated that it exhibited a melting endotherm at approximately 180 °C. 1:5 ondansetron-cyclodextrin complexes showed a wide endothermic peak indicative of water loss usually observed in amorphous form and a small endothermic peak at around 180 °C which is the melting point of ondansetron (Figure 4). Different drug-cyclodextrin complexes were tested in the study 1:1, 1:5, 1:20 to optimise the preparation of the complexes to get the best inclusion. The ratio 1:1 showed crystalline form of the drug indicating that most of the drug was not included in the CD complexes (Data not shown). The 1:5 and 1:20 ratios showed very low and no crystalline form of ondansetron respectively indicating that most of the drug was incorporated in the drug-cyclodextrin complexes or in an amorphous state (Figure 4, shows DSC data from 1:5 ondansetron-cyclodextrin). The ratio 1:5 ondansetron-cyclodextrin was selected to prepare the printlets because it offers the best proportion to obtain the right dose of the drug in the printlets without increasing considerably the amount of excipients (CD) required. Although a small part of the drug may be not incorporated into the cyclodextrin, drug-cyclodextrin complexes may be formed “in situ” in the mouth facilitated by the saliva as a solvent. The DSC data of the formulations before and after printing showed a sharp endothermic peak at around 168 °C which corresponds to the melting point of the mannitol. The absence of the endothermic peak corresponding to the melting point of the ondansetron indicates that the drug is in the amorphous form within the formulations or that the drug percentage is so low that the crystals (if any) are not detected using DSC.

X-ray diffractograms do not provide clear information and cannot be used to confirm the results from the DSC (Figure 5). XRPD patterns of the individual components show that mannitol and the drug-cyclodextrin complexes have some peaks corresponding to crystalline forms. Kollidon VA 64 showed wide halos indicative of the amorphous form. Crystalline ondansetron peaks are not observed in the drug-cyclodextrin complexes incorporated into the formulations due to the crystallinity of mannitol. XRPD patterns of the formulations both before and after printing showed sharp peaks which are indicative of a crystalline form of mannitol but do not provide useful information about the state of the drug and how it is incorporated into the polymers.

**Figure 6:**
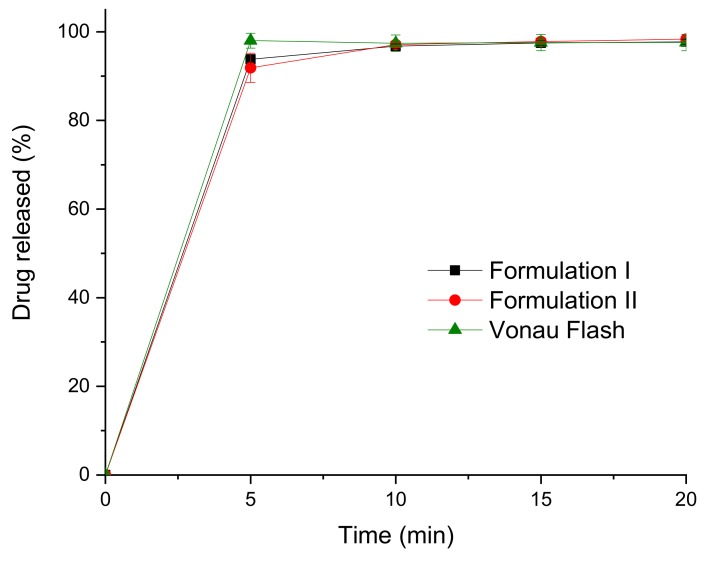
Dissolution profiles of the commercial and the 3D printed formulations.

Drug release profiles from the printlets were obtained using a USP II dissolution test (Figure 6). Drug dissolution profiles for both formulations show that the drug is almost completely dissolved in around 5 min, the formulations disintegrated and dissolved so fast that conventional USP II dissolution tests are not useful in comparing these formulations.

The disintegration time of the printlets was determined using the compendial disintegration equipment. The printlets completely disintegrated in around 15 s (Table 2), which agrees with the dissolution profiles. The disintegration times are in line with the values of the commercial formulation (14.3 ± 2.7 s). As the disintegration time is lower than 30 s, these printlets would be considered ODTs according to the European Pharmacopoeia and the FDA.

The increment of the percentage of mannitol (60% *w/w*) in Formulation II compared to Formulation I did not change significantly the mechanical characteristics of the printlets although it was expected to reduce the mechanical properties and increase the drug release rate by reducing the disintegration time. Only a low percentage of Kollidon VA-64 (15% *w/w*) was enough to obtain the 3D printed formulations and maintain the structure of the printlets. The reduction of the percentage of Kollidon VA-64, which is the polymer that maintains the structure, to only 15% *w/w* in formulations II allows the use of 82% *w/w* for other materials like drugs (allowing higher drug loading) or excipients like mannitol or cyclodextrins (for taste masking effects).

The results confirm that SLS 3D printing technology can be a suitable technique for the manufacture of ODPs incorporating cyclodextrins. The rapid disintegration time makes these formulations comparable to commercial ODT formulations or to formulations prepared by powder bed inkjet 3D printing formulations already available in the market [58].

SLS technology can be used with a wide variety of excipients, modifying the drug release profile of formulations and transforming them to be amenable for ODP formulations. SLS 3D printing has the potential to be scaled up in a similar way to powder bed inkjet 3D printing, without the potential issue of using water in the process. The technology could be also adapted to produce 3D printed formulations at the point of dispensing as the printlets manufactured by the solvent-free process can by readily dispensable and would not require an additional drying step following printing. The opportunity to manufacture this drug product in an automatic manner close to the point of dispensing opens new opportunities in the implementation of personalised medicine as there is the need for automatic, cost-effective and reliable systems to prepare oral medicines personalised to the individual.

## 4. Conclusions

SLS 3D printing was used to manufacture orally disintegrating 3D printed printlets of two formulations of ondansetron. The formulations included ondansetron in drug-cyclodextrin complexes and a high percentage of mannitol (up to 60%) to improve taste masking. Both printlets types showed fast disintegration (~15 s) and released more than 90% of the drug in 5 min independent of the mannitol content. This work demonstrates the potential of SLS 3DP to fabricate orodispersible printlets comparable in disintegration time and drug release rate to a commercial ODT using a manufacturing technology amenable to the preparation of personalised dose medicines.

## Figures and Tables

**Table 1:** Composition of the formulations (*w/w*).

Formulation	1:5 Ondansetron: Cyclodextrin Complex	Kollidon VA-64	Mannitol	Candurin^®^ Gold Sheen
Formulation-I	22%	25%	50%	3%
Formulation-II	22%	15%	60%	3%

**Table 2:** Characteristics of the formulations.

Formulation	Mean Mass ± SD (mg)	Diameter ± SD (mm)	Height ± SD (mm)	Breaking Force (N)	% Drug Loading from Theoretical Content ± SD (%)	Disintegration Time ± SD (s)
Formulation I	217.2 ± 4.2	11.7 ± 0.1	4.4 ± 0.2	14.7 ± 2.5	98.6 ± 2.2	14.3 ± 3.1
Formulation II	211.3 ± 7.3	11.9 ± 0.1	3.7 ± 0.1	18.5 ± 5.0	98.1 ± 1.7	15.3 ± 2.3
